# Supplementary material for: Succinylation of a KEAP1 sensor lysine promotes NRF2 activation
Source: bioRxiv. 2023 May 9:2023.05.08.539908. Preprint. [Version 1] doi: 10.1101/2023.05.08.539908 (PMC10197519; doi:10.1101/2023.05.08.539908)
Supplement: Supplement 1 [file NIHPP2023.05.08.539908v1-supplement-1.pdf]

bioRxiv preprint doi: https://doi.org/10.1101/2023.05.08.539908; this version posted May 9, 2023. The copyright holder for this preprint (which was not certified by peer review) is the author/funder, who has granted bioRxiv a license to display the preprint in perpetuity. It is made available under aCC-BY 4.0 International license.

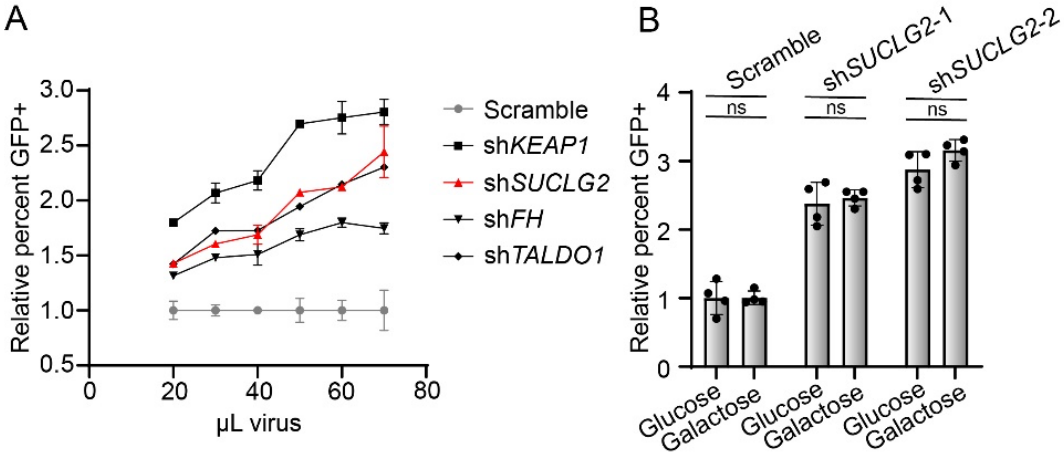

Supplementary Figure 1. Knockdown of *SUCLG2* activates ARE-GFP-LUC. A) Relative ARE activation from ARE-GFP-LUC K562 cells in response to the indicated volumes of lentiviruses encoding the indicated shRNAs. B) Relative ARE activation from ARE-GFP-LUC K562 cells in response to treatment with lentiviruses encoding the indicated shRNAs to *SUCLG2* grown in either glucose or galactose (*n*=4; ns = not significant *P*>0.05, two-way ANOVA).

bioRxiv preprint doi: https://doi.org/10.1101/2023.05.08.539908; this version posted May 9, 2023. The copyright holder for this preprint (which was not certified by peer review) is the author/funder, who has granted bioRxiv a license to display the preprint in perpetuity. It is made available under aCC-BY 4.0 International license.

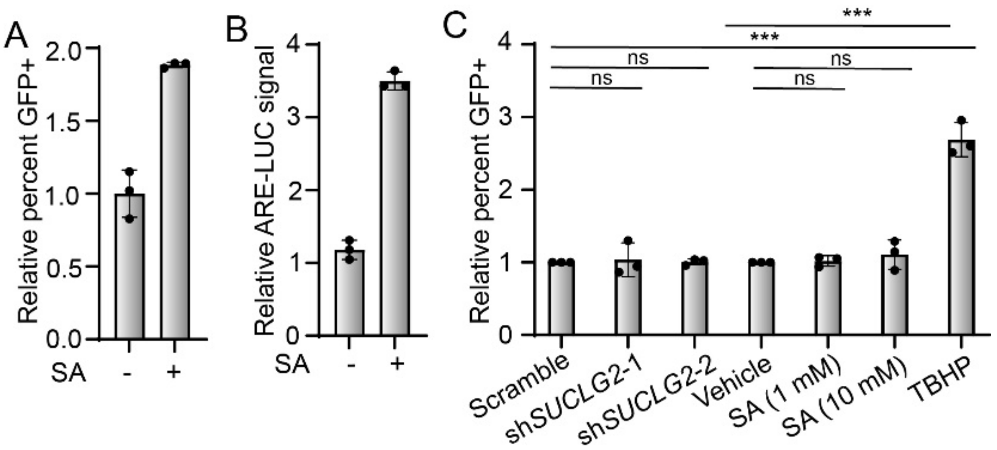

Supplementary Figure 2. SA activates ARE-GFP-LUC in K562 cells. A) Relative percent GFP positive K562 ARE-GFP-LUC cells after treatment with 50 mM SA for 4 hrs (*n*=3, ***P<0.001, t-test). B) Relative luminescence signal of reporter activity from K562 ARE-GFP-LUC cells after treatment with 50 mM SA for 4 hrs (*n*=3, ***P<0.001, t-test). C) Relative percent GFP positive measurements indicating the presence of reactive species from K562 cells treated with virus expressing shRNAs targeting *SUCLG2* or treatment with the indicated compounds (TBHP = tert-butyl hydro peroxide; *n*=3, ns = not significant P>0.05, ****P<0.0001, one-way ANOVA).

bioRxiv preprint doi: https://doi.org/10.1101/2023.05.08.539908; this version posted May 9, 2023. The copyright holder for this preprint (which was not certified by peer review) is the author/funder, who has granted bioRxiv a license to display the preprint in perpetuity. It is made available under aCC-BY 4.0 International license.

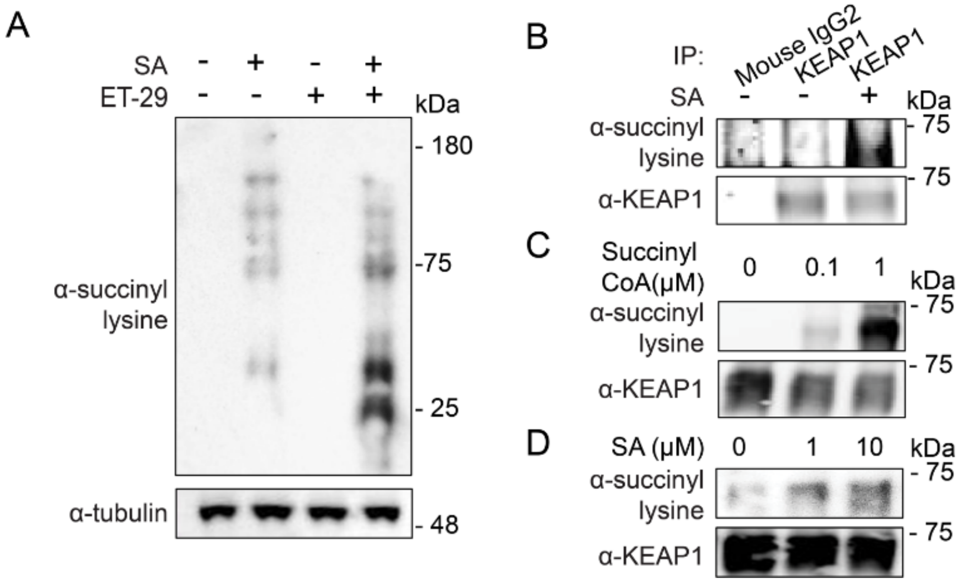

Supplementary Figure 3. Covalent modification of KEAP1 lysines in response to SA. A) Western blotting analysis for anti-succinyl lysine positivity after treatment of HEK293T cells with ET-29 (10 μM) and SA (5 mM, 1 hr). B) Western blotting anti-succinyl lysine positivity from pulldowns of endogenous KEAP1 from HEK293T cells treated with SA (5 mM, 1 hr). Western blotting anti-succinyl lysine positivity of recombinant KEAP1 after a 1 hr in vitro treatment with the indicated concentrations of Succinyl-CoA (C) or SA (D).

bioRxiv preprint doi: https://doi.org/10.1101/2023.05.08.539908; this version posted May 9, 2023. The copyright holder for this preprint (which was not certified by peer review) is the author/funder, who has granted bioRxiv a license to display the preprint in perpetuity. It is made available under aCC-BY 4.0 International license.

#	b	b^{++}	b*	b^{*++}	b^{0}	b^{0++}	Seq.	y	y^{++}	y*	y^{*++}	y^{0}	y^{0++}	#
1	130.0499	65.5286			112.0393	56.5233	E							19
2	258.1084	129.5579	241.0819	121.0446	240.0979	120.5526	Q	2139.0573	1070.0323	2122.0307	1061.519	2121.0467	1061.027	18
3	315.1299	158.0686	298.1034	149.5553	297.1193	149.0633	G	2010.9987	1006.003	1993.9722	997.4897	1992.9881	996.9977	17
4	446.1704	223.5888	429.1438	215.0756	428.1598	214.5836	M	1953.9772	977.4923	1936.9507	968.979	1935.9667	968.487	16
5	575.213	288.1101	558.1864	279.5969	557.2024	279.1049	E	1822.9368	911.972	1805.9102	903.4587	1804.9262	902.9667	15
6	674.2814	337.6443	657.2549	329.1311	656.2708	328.6391	V	1693.8942	847.4507	1676.8676	838.9374	1675.8836	838.4454	14
7	773.3498	387.1785	756.3233	378.6653	755.3393	378.1733	V	1594.8258	797.9165	1577.7992	789.4032	1576.8152	788.9112	13
8	860.3818	430.6946	843.3553	422.1813	842.3713	421.6893	S	1495.7573	748.3823	1478.7308	739.869	1477.7468	739.377	12
9	973.4659	487.2366	956.4394	478.7233	955.4553	478.2313	I	1408.7253	704.8663	1391.6988	696.353	1390.7147	695.861	11
10	1102.5085	551.7579	1085.482	543.2446	1084.4979	542.7526	E	1295.6412	648.3243	1278.6147	639.811	1277.6307	639.319	10
11	1159.53	580.2686	1142.5034	571.7553	1141.5194	571.2633	G	1166.5987	583.803	1149.5721	575.2897	1148.5881	574.7977	9
12	1272.614	636.8107	1255.5875	628.2974	1254.6035	627.8054	I	1109.5772	555.2922	1092.5506	546.779	1091.5666	546.287	8
13	1409.6729	705.3401	1392.6464	696.8268	1391.6624	696.3348	H	996.4931	498.7502	979.4666	490.2369	978.4826	489.7449	7
14	1506.7257	753.8665	1489.6992	745.3532	1488.7151	744.8612	P	859.4342	430.2207	842.4077	421.7075	841.4237	421.2155	6
15	1734.8367	867.922	1717.8102	859.4087	1716.8261	858.9167	K	762.3815	381.6944	745.3549	373.1811	744.3709	372.6891	5
16	1833.9051	917.4562	1816.8786	908.9429	1815.8946	908.4509	V	534.2704	267.6389	517.2439	259.1256	516.2599	258.6336	4
17	1964.9456	982.9764	1947.9191	974.4632	1946.935	973.9712	M	435.202	218.1047	418.1755	209.5914	417.1915	209.0994	3
18	2093.9882	1047.4977	2076.9617	1038.9845	2075.9776	1038.4925	E	304.1615	152.5844	287.135	144.0711	286.151	143.5791	2
19							R	175.119	88.0631	158.0924	79.5498			1

Supplementary Figure 4. Lysine 131 is succinylated by SA in cells. *b* and *y* ion designations corresponding to MS/MS spectra in Figure 4A.

bioRxiv preprint doi: https://doi.org/10.1101/2023.05.08.539908; this version posted May 9, 2023. The copyright holder for this preprint (which was not certified by peer review) is the author/funder, who has granted bioRxiv a license to display the preprint in perpetuity. It is made available under aCC-BY 4.0 International license.

A

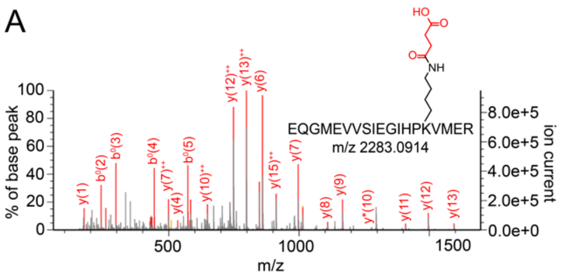

#	b	b⁺⁺	b*	b*⁺⁺	b⁰	b⁰⁺⁺	Seq.	y	y⁺⁺	y*	y*⁺⁺	y⁰	y⁰⁺⁺	#
1	130.0499	65.5286			112.0393	56.5233	E							19
2	258.1084	129.5579	241.0819	121.0446	240.0979	120.5526	Q	2155.0522	1078.0297	2138.0257	1069.5165	2137.0416	1069.0245	18
3	315.1299	158.0686	298.1034	149.5553	297.1193	149.0633	G	2026.9936	1014.0005	2009.9671	1005.4872	2008.9831	1004.9952	17
4	462.1653	231.5863	445.1388	223.073	444.1547	222.581	M	1969.9722	985.4897	1952.9456	976.9764	1951.9616	976.4844	16
5	591.2079	296.1076	574.1814	287.5943	573.1973	287.1023	E	1822.9368	911.972	1805.9102	903.4587	1804.9262	902.9667	15
6	690.2763	345.6418	673.2498	337.1285	672.2658	336.6365	V	1693.8942	847.4507	1676.8676	838.9374	1675.8836	838.4454	14
7	789.3447	395.176	772.3182	386.6627	771.3342	386.1707	V	1594.8258	797.9165	1577.7992	789.4032	1576.8152	788.9112	13
8	876.3768	438.692	859.3502	430.1787	858.3662	429.6867	S	1495.7573	748.3823	1478.7308	739.869	1477.7468	739.377	12
9	989.4608	495.2341	972.4343	486.7208	971.4503	486.2288	I	1408.7253	704.8663	1391.6988	696.353	1390.7147	695.861	11
10	1118.5034	559.7553	1101.4769	551.2421	1100.4929	550.7501	E	1295.6412	648.3243	1278.6147	639.811	1277.6307	639.319	10
11	1175.5249	588.2661	1158.4983	579.7528	1157.5143	579.2608	G	1166.5987	583.803	1149.5721	575.2897	1148.5881	574.7977	9
12	1288.6089	644.8081	1271.5824	636.2948	1270.5984	635.8028	I	1109.5772	555.2922	1092.5506	546.779	1091.5666	546.287	8
13	1425.6679	713.3376	1408.6413	704.8243	1407.6573	704.3323	H	996.4931	498.7502	979.4666	490.2369	978.4826	489.7449	7
14	1522.7206	761.8639	1505.6941	753.3507	1504.7101	752.8587	P	859.4342	430.2207	842.4077	421.7075	841.4237	421.2155	6
15	1750.8316	875.9195	1733.8051	867.4062	1732.8211	866.9142	K	762.3815	381.6944	745.3549	373.1811	744.3709	372.6891	5
16	1849.9	925.4537	1832.8735	916.9404	1831.8895	916.4484	V	534.2704	267.6389	517.2439	259.1256	516.2599	258.6336	4
17	1980.9405	990.9739	1963.914	982.4606	1962.93	981.9686	M	435.202	218.1047	418.1755	209.5914	417.1915	209.0994	3
18	2109.9831	1055.4952	2092.9566	1046.9819	2091.9726	1046.4899	E	304.1615	152.5844	287.135	144.0711	286.151	143.5791	2
19							R	175.119	88.0631	158.0924	79.5498			1

B

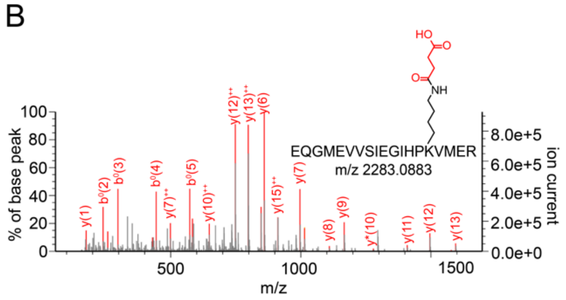

#	b	b⁺⁺	b*	b*⁺⁺	b⁰	b⁰⁺⁺	Seq.	y	y⁺⁺	y*	y*⁺⁺	y⁰	y⁰⁺⁺	#
1	130.0499	65.5286			112.0393	56.5233	E							19
2	258.1084	129.5579	241.0819	121.0446	240.0979	120.5526	Q	2155.0522	1078.0297	2138.0257	1069.5165	2137.0416	1069.0245	18
3	315.1299	158.0686	298.1034	149.5553	297.1193	149.0633	G	2026.9936	1014.0005	2009.9671	1005.4872	2008.9831	1004.9952	17
4	462.1653	231.5863	445.1388	223.073	444.1547	222.581	M	1969.9722	985.4897	1952.9456	976.9764	1951.9616	976.4844	16
5	591.2079	296.1076	574.1814	287.5943	573.1973	287.1023	E	1822.9368	911.972	1805.9102	903.4587	1804.9262	902.9667	15
6	690.2763	345.6418	673.2498	337.1285	672.2658	336.6365	V	1693.8942	847.4507	1676.8676	838.9374	1675.8836	838.4454	14
7	789.3447	395.176	772.3182	386.6627	771.3342	386.1707	V	1594.8258	797.9165	1577.7992	789.4032	1576.8152	788.9112	13
8	876.3768	438.692	859.3502	430.1787	858.3662	429.6867	S	1495.7573	748.3823	1478.7308	739.869	1477.7468	739.377	12
9	989.4608	495.2341	972.4343	486.7208	971.4503	486.2288	I	1408.7253	704.8663	1391.6988	696.353	1390.7147	695.861	11
10	1118.5034	559.7553	1101.4769	551.2421	1100.4929	550.7501	E	1295.6412	648.3243	1278.6147	639.811	1277.6307	639.319	10
11	1175.5249	588.2661	1158.4983	579.7528	1157.5143	579.2608	G	1166.5987	583.803	1149.5721	575.2897	1148.5881	574.7977	9
12	1288.6089	644.8081	1271.5824	636.2948	1270.5984	635.8028	I	1109.5772	555.2922	1092.5506	546.779	1091.5666	546.287	8
13	1425.6679	713.3376	1408.6413	704.8243	1407.6573	704.3323	H	996.4931	498.7502	979.4666	490.2369	978.4826	489.7449	7
14	1522.7206	761.8639	1505.6941	753.3507	1504.7101	752.8587	P	859.4342	430.2207	842.4077	421.7075	841.4237	421.2155	6
15	1750.8316	875.9195	1733.8051	867.4062	1732.8211	866.9142	K	762.3815	381.6944	745.3549	373.1811	744.3709	372.6891	5
16	1849.9	925.4537	1832.8735	916.9404	1831.8895	916.4484	V	534.2704	267.6389	517.2439	259.1256	516.2599	258.6336	4
17	1980.9405	990.9739	1963.914	982.4606	1962.93	981.9686	M	435.202	218.1047	418.1755	209.5914	417.1915	209.0994	3
18	2109.9831	1055.4952	2092.9566	1046.9819	2091.9726	1046.4899	E	304.1615	152.5844	287.135	144.0711	286.151	143.5791	2
19							R	175.119	88.0631	158.0924	79.5498			1

C

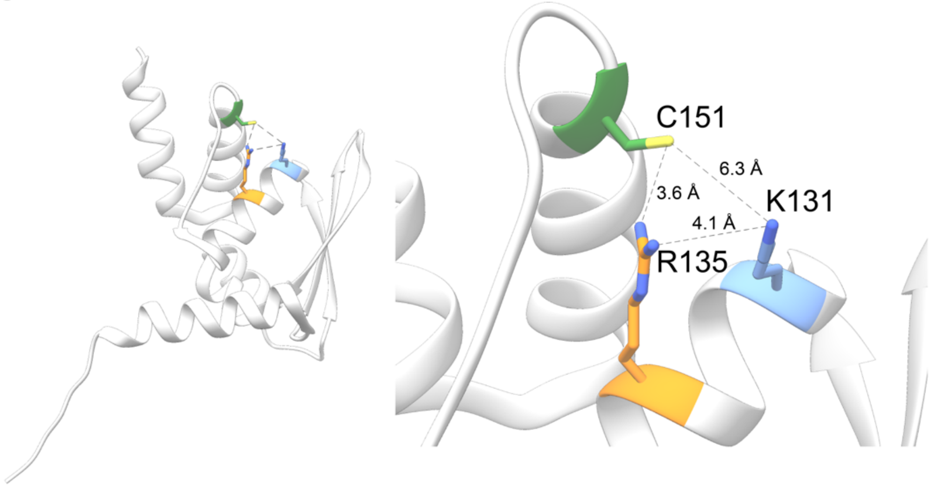

D

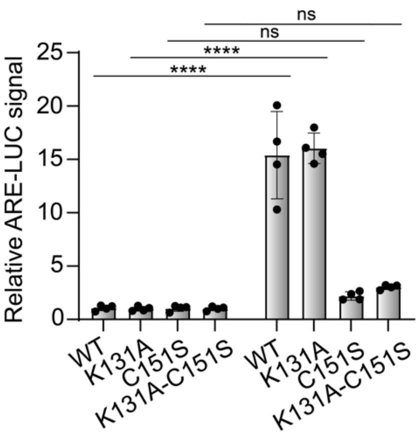

Supplementary Figure 5: Lysine 131 of KEAP1 is covalently modified by SA. MS/MS spectra (left) along with *b* and *y* ion designations (right) corresponding to +101 Da modified tryptic KEAP1 peptides K131 from 1 hr *in vitro* reactions of SA (10 mM (A) and 1 mM (B)) with recombinant KEAP1. C) Representation of the BTB domain of KEAP1 (left; PDB: 4CXI) with inset denoting orientations of key residues K131, R135, and C151(right). D) ARE-luciferase reporter activity of IMR32 cells expressing the indicated KEAP1 transgene mutants after treatment with 500 nM bardoxolone methyl for 24 hrs (*n*=4, ns = not significant P>0.05, ****P<0.0001, two-way ANOVA).
